# Using the capability, opportunity, and motivation model of behaviour to assess provider perception of implementing solution-focused goal-setting in paediatric rehabilitation

**DOI:** 10.1177/13674935231194501

**Published:** 2023-08-16

**Authors:** L Crawford, H Colquhoun, S Kingsnorth, D Fehlings, Nora Fayed

**Affiliations:** 1 School of Rehabilitation Science, Queen’s University, Kingston, ON, Canada; 2 Department of Occupational Science and Occupational Therapy, University of Toronto, Toronto, ON, Canada; 3 Rehabilitation Sciences Institute, University of Toronto, Toronto, ON, Canada; 4 Bloorview Research Institute, Toronto, ON, Canada; 5 Department of Paediatrics, Faculty of Medicine, University of Toronto, Toronto, ON, Canada; 6 Health Services and Policy Research Institute, Queen’s University, Kingston, ON, Canada

**Keywords:** Goals, implementation science, professional-patient relations, child

## Abstract

Adoption of family and child goal-setting in paediatric rehabilitation is important to positive long-term outcomes. Solution-focused coaching (SFC) has been identified as a promising approach to ensuring this type of goal-setting occurs, while the actual implementation of SFC by health care providers (HCPs) is low. This study utilized the capacity, opportunity, and motivation model of behaviour change (COM-B) to identify which strengths and difficulties health care providers (HCPs) perceived with respect to SFC goal-setting in paediatric rehabilitation. A self-report survey was developed and administered to HCPs at a paediatric rehabilitation hospital. Each survey question was based upon a COM-B sub-component. Demographic information was collected from HCPs, and descriptive statistics were used to rank perceived COM-B components from strongest to weakest. Results indicate HCPs view the provision of SFC goal-setting as an important practice, while they also perceive difficulties to actual delivery due to: lack of adequate individual skill, lack of experience with this type of goal-setting, and insufficient preparation for clients to engage in sharing their goals. HCPs also perceived lack of organizational processes to support the practice within their teams. Recommendations for intervention are provided.

Setting #child and #family-centred #goals in #pedsrehab is challenging.

We learned from #HCPs that they support family goals but want more mentorship and routines to make it happen all the time.

## Introduction

Over the last 20 years, there has been a growing focus on embedding goal-setting into rehabilitation as a critical component of family centred practice (Coyne et al., 2016; Uniacke et al., 2018). Shared goal-setting between health care providers (HCPs) and families ensures that objectives for rehabilitation are identified by people who know the child well and tailored each child’s well-being (Baldwin et al., 2013; King et al., 2018; King et al., 2019; Seko et al., 2020; Schwellnus et al., 2020b). Adoption of formalized goal-setting is rarely implemented in rehabilitation practice despite its importance to child and family centred care (Plant et al., 2016; Rosewilliam et al., 2011; Sugavanam et al., 2013).

Solution-focused coaching (SFC) has been identified as a promising approach to ensure that child and family centred goal-setting occurs (Baldwin et al., 2013; King et al., 2019; Schwellnus et al., 2020a; Seko et al., 2021). The approach emphasizes ‘how’ to work collaboratively with families to enhance the well-being and participation of children with disabilities (King et al., 2019; Robinson et al., 2011; Schwellnus et al., 2020a; Seko et al., 2021). An important first step in SFC is for HCPs to identify goals in collaboration with each child and family that that fits their unique environment, developmental stage, and ability. SFC is a strengths-based approach that encourages clients to set goals and identify their preferred future while leveraging their strengths, skills, and resources to build on what is already working in their lives (Berg and Szabo, 2005; De Shazer et al., 1986; Ratner and Yusuf, 2015). This approach supports child and family goal attainment, confidence in skills and abilities, and self-determination (Baldwin et al., 2013; King et al., 2003, 2018; McPherson et al., 2018, 2019; Schwellnus et al., 2020a).

Despite the promise of SFC goal-setting within rehabilitation, its use has been limited among individual providers and multidisciplinary teams to date (Seko et al., 2020; Seko et al., 2021; Schwellnus et al., 2020b). Evidence shows that HCPs need to be the focus of behaviour change interventions to promote adoption of a client-centred, collaborative goal-setting approach (McDonagh et al., 2018; Plant et al., 2016; Seko et al., 2020, 2021; Schwellnus et al., 2020b). Barriers to goal-setting have been identified (Levack et al., 2006a, 2006b, 2015; Plant et al., 2016; Rosewilliam et al., 2011; Sugavanam et al., 2013), but there has been little empirical research quantifying which specific behavioural components HCPs perceive are impeding their adoption. Understanding HCP perceptions is important to planning which components to address in a behaviour change intervention directed at them.

In this study, the Capability, Opportunity, and Motivation Model of Behaviour (COM-B) will be used to examine HCP perceptions of SFC goal-setting (Michie et al., 2014). The COM-B model espouses that there are three overarching components: capability, opportunity, and motivation that interact to promote or hinder behaviour change. In this case, ‘capability’ represents an HCP’s psychological (e.g. knowledge) or physical (e.g. skills) capacity to set child or family centred goals; opportunity represents social (e.g. societal influences) or physical (e.g. environmental resources or context) factors that make it possible for an HCP to set these collaborative goals; while motivation will represent the automatic (e.g. emotions) or reflective (e.g. beliefs about capabilities or intentions) cognitive processes that drive the HCP to set goals in the SFC framework (Michie et al., 2014). The COM-B model has been effectively applied to many health behaviours at both individual and organizational levels (Barker et al., 2016; McDonagh et al., 2018) but has not yet been applied to assess HCPs use of SFC goal-setting. In this descriptive study, a survey of HCPs was conducted using the COM-B model to understand their perceptions of the application of SFC goal-setting in paediatric rehabilitation practice.

### Aim

To use the COM-B model to explore which components of behaviour change, HCPs perceive as strengths or difficulties to the delivery of SFC goal-setting in their own paediatric rehabilitation practice.

## Methods

Ethical approval for this study was granted by the Research Ethics Boards of Bloorview Kid’s Rehabilitation Hospital (REB#0121) and Queen’s University (REB#6030001), publicly funded through provincial social and healthcare ministries, mandated to serve any child with a functional physical limitation irrespective of the diagnostic category. Basic SFC training was introduced from December 2016 to December 2018, provided by a certified SFC trainer to all HCPs, administrative leaders (e.g. program managers), and clinical students/trainees in the form of a mandatory 2-day (12 h) workshop. Non-HCP staff who had patient contact (e.g. family liaisons or volunteers) were also invited to attend, although training was not mandatory for them. Workshops included an overview of SFC instruction and examples of SFC goal-setting, co-construction of action plans, and active listening (Baldwin et al., 2013; King et al., 2018, 2019; Schwellnus et al., 2020a, 2020b; Seko et al., 2020, 2021).

Following the initial training, providers were encouraged to take part in non-mandatory additional practice activities (2019–2021), which included online modules, team practice sessions, individual coaching, and lunch and learns. Two certificate programs for HCPs, titled: ‘Certified Solution-Focused Health Care Coach’ and ‘Solution-Focused Facilitation’ were also offered, but HCP participation was not tracked or monitored by the organization.

### Study question and design

What did HCPs perceive as strengths and difficulties to the delivery of SFC goal-setting in paediatric rehabilitation?

#### Study survey

The authors (LC, NF) developed a 64-item web-based self-report survey based on the results from a review of the barriers and facilitators to patient-centred goal-setting (Crawford et al., 2022). The items were organized to align with the COM-B model components and sub-components (Michie et al., 2014). ‘**Capability-(psychological)’** included perceived knowledge of SFC goal-setting, cognitive and interpersonal skills to set goals, memory attention and decision processes, and behavioural regulation capacity. ‘**Capability-(physical)’** included HCPs perceived skills to identify and implement child or family goals in practice. ‘**Opportunity-(social)’** included items about the HCP’s perception of social influences (e.g. team members/managers) on SFC goal-setting, while ‘**opportunity-(physical)’** items were about the context in which HCPs set goals with clients, and the resources HCPs believe are available to support identifying and implementing goals. ‘**Motivation-(reflective)’** included the cognitive processes HCPs use to plan to engage in goal-setting, while **‘motivation-(automatic)’** involved unconscious professional role-identity, beliefs about capabilities and consequences, optimism, intentions, and emotions that drive HCPs to set these child and family centred goals (Michie et al., 2014).

An initial version of the survey was independently reviewed by five HCP-SFC experts to ensure item comprehension and improve content validity of the items (Creswell and Creswell, 2018). Modifications of the survey based on feedback from the HCP-SFC experts included: the re-organization of some items relative to COM-B components, the consistency of terms used in the items, simplified language, and brief examples to clarify the intent of items. The final survey and the organization of items relative to COM-B components are included in Supplement A.

Examples of items arranged by the COM-B components were ‘capability-(psychological)’: ‘I know what SFC is and know how to use it in practice’; ‘opportunity-(physical)’: ‘I have the time, space and supports to deliver SFC goal-setting’; and ‘motivation-(reflective)’: ‘I feel like SFC goal-setting is aligned with my therapeutic processes’ (Michie et al., 2014).

The survey was administered via REDCap© (https://www.redcap.com), with an estimated 30 min to completion time, using a 5-point scale, which assessed the extent to which HCPs agreed or disagreed with each survey statement from ‘1- strongly disagree’ to ‘5- strongly agree’) (Supplement A – Survey Questions).

### Population

Any HCPs within the hospital who had opportunity to conduct goal-setting with clients and families (e.g. occupational therapists, physiotherapists, social workers, early childhood educators, psychologists, nurses, physicians, and life skill coaches) were eligible for the study including those who were new to the organization who were not present for the mandatory SFC training provided prior to 2018. Managerial staff without any patient contact in the year preceding the survey and staff with no patient contact were excluded.

### Data collection

An email invitation was directly sent to potential participants, which contained a web-based consent and link to the survey. The survey was available for completion from September 2020 to June 2021. Reminder emails were sent in January and April 2021. Demographic information including profession-type, years of experience, years worked at the organization, area of practice, and amount and type of SFC training were collected.

### Data analysis

Demographic information was analyzed descriptively and reported for variables of profession, program area, amount of SFC experience, and education. To describe and then rank the perceived strengths and difficulties within each COM-B component, mean scores were used as the point estimate of each SFC barrier or facilitator, with standard deviation as the indicator of variance. Response distributions of all items were plotted via histograms. Negatively phrased items were reverse-scored so that higher scores (from one to five) could be interpreted as strengths to SFC implementation and lower scores as difficulty to SFC implementation. No COM-B component (i.e. survey item) was decided a priori as a facilitator or barrier to implementation. Instead, the ranking of COM-B components relative to each other was the focus of the interpretation of study findings. Internal-consistencies were not performed for items within the COM-B components because summary scores were not justified. Item mean scores were only meaningful relative to other item scores, not as stand-alone scores.

## Results

The online survey was sent to 230 HCPs and 134 responses were received (58% response rate). Fifty-eight HCPs (%) provided consent but did not provide survey responses aside from demographic data, hence, they were omitted from the analysis. Overall, 76 (33%) surveys were completed. A summary of participant demographic data is provided in Table 1. Profession of respondents was consistent with the composition of professions that form the staff in the hospital (Table 1), with the exception of therapeutic recreation, which form a smaller staff group than physical therapy or nursing but were represented more heavily in the study. Settings represented were primarily from outpatient services 51 (68%) with an even split between the medical clinics and rehabilitation and recreational programs. In-patient HCPs 21 (28%) were mostly from the brain injury and orthopaedic teams, which are consistent with the bulk of in-patient services. The majority of participants 48 (63%) had over 11 years of clinical experience. 10 participants (27%) were undertaking the advanced SFC certificate training to become a certified SF-coach.

**Table 1.** Participant demographics *N* = 76.

Health professional discipline	*n* (%)
Occupational therapist	13 (17)
Therapeutic recreation	13 (17)
Physical therapist	10 (13)
Rehabilitation assistant	10 (13)
Speech language pathologist	8 (11)
Registered nurse	8 (11)
Social worker	4 (5)
Psychologist	3 (4)
Physician	2 (3)
Other (pharmacist, spiritual care advisor)	2 (2)
Missing	3 (4)
Clinical program area	*n* (%)
**Outpatient programs**	**51 (68)**
Child development	29 (39)
Participation and inclusion	22 (29)
**In-patient units**	**21 (28)**
Brain injury	12 (16)
Specialized orthopaedic	8 (11)
Complex continuing care	1 (1)
Missing	3 (4)
Years of professional experience	
0–2	4 (5)
3–5	8 (11)
6–10	16 (21)
11–20	34 (45)
Over 20	14 (18)
Formal SFC training – # of hours	*n* (%)
None	5 (7)
1–10 hours	27 (36)
11–25	30 (39)
Greater than 25	14 (18)
SFC Training type	*n* (%)
Organizational training (12 h)	60 (79)
External course/conference	32 (43)
Individual coaching	23 (30)
Guest speaker	14 (18)
Solution focused health care coach certification	10 (13)

### Capacity

HCPs highly endorsed items related to general knowledge and understanding of SFC components of goal-setting (Table 2). Lower-scored items related to: specific training to enact the goals in intervention or confidence in shared goal-setting with the children themselves.

**Table 2.** Mean scores and ranking of perceived COM-B capability.

Ranking*	Capability sub-component	Item		Mean (SD)**
1	Psychological	HCP goals support participation in real world activities		4.3 (0.7)
2	Psychological and physical	HCP has knowledge of, and knows how to use SFC goal-setting in practice		4.0 (0.8)
3	Psychological	HCP formulates goals based on child’s concerns		3.9 (0.7)
4	Psychological	HCP formulates goals on client’s desired future		3.9 (0.7)
5	Psychological	Goals are activity/play based		3.9 (0.9)
5	Psychological	HCP formulates goals on parent’s concerns		3.9 (0.7)
7	Psychological	HCP formulates goals on parent’s desired future		3.9 (0.7)
8	Psychological	HCP know how to conduct SFC goal-setting with parents		3.8 (0.9)
9	Psychological	HCP understands assessment components		3.6 (0.9)
9	Psychological	HCP understands intervention components		3.6 (0.9)
11	Psychological and physical	HCP is proficient with clients		3.6 (0.8)
12	Psychological	HCP is competent using with parents		3.5 (1.0)
13	Psychological	HCP is competent using with clients		3.4 (0.9)
14	Physical	HCP adequately trained to deliver with parents		3.4 (1.0)
15	Physical	HCP adequately trained to deliver with clients		3.4 (0.9)
16	Psychological	HCP is confident using SFC goal-setting with children		3.4 (1.0)
17	Psychological	HCP bases goals on past problems		2.9 (0.9)

*Higher ranking indicates higher perceived level of capability by HCP with SFC goal-setting. **All items scored from 1 to 5, where 5 indicates a most positive level of the attribute.

### Opportunity

Within this domain, HCPs highest scored items related to effectiveness and organizational opportunities for SFC goal-setting with lower endorsement that children, families, and teams expected this form of care (Table 3). Physical opportunity barrier results indicated a lack of time, human resources, and clinical processes (e.g. documentation) to support the practice.

**Table 3.** Mean scores and ranking of perceived COM-B opportunity.

Ranking^a^	Opportunity sub-component	Item	Mean (SD)^b^
1	Social	Improves child’s outcomes	3.9 (0.7)
2	Social	Organization supports	3.8 (0.9)
3	Social	Improves parent outcomes	3.8 (0.7)
4	Social	Colleague’s support	3.7 (0.9)
5	Physical	Appropriate space	3.6 (0.9)
6	Social	Families support use	3.6 (0.7)
7	Social	Organizational expectation to use	3.3 (1.0)
8	Physical	Adequate human resources	3,2 (0.9)
8	Social	Clinical team expectation	3.2 (1.0)
10	Physical	Adequate time (clinician)	3.1 (1.0)
11	Physical	Documentation supports	3.0 (1.0)
12	Physical	Adequate time (team)	2.9 (1.0)
12	Social	Adequate team processes	2.9 (1.0)
14	Social	Expectation for families	2.6 (0.7)

^a^Higher ranking indicates higher perceived level of opportunity by HCP with SFC goal-setting.

^b^All items scored from 1 to 5, where 5 indicates a most positive level of the attribute.

### Motivation

The highest mean scores in the motivation domain demonstrated HCPs believe that clients are experts in their lives, and they integrate needs and preferences of the child and family into goal-setting (Table 4). The top half-ranked items in this component relate to beliefs that SFC is aligned with professional identity, that SFC is complementary to the therapeutic process, it aligned with the clinical team values, and its use promotes motivation and positive emotions. Items ranked in the middle of the motivation sub-component were associated with HCP anxiety or loss of control in goal-setting, suggesting that some clinicians experience negative emotions and others did not. Lowest-ranked items related to HCP confidence to conduct and deliver SFC goal-setting as well as perceived lack of team and organizational support.

**Table 4.** Mean scores and ranking of perceived COM-B motivation.

Ranking^a^	Motivation sub-component	Item	Mean (SD)^b^
1	Reflective	Listen to child’s wishes	4.6 (0.5)
2	Reflective	Children experts in lives	4.6 (0.6)
3	Reflective	Client wishes integrated	4.5 (0.6)
4	Reflective	Parent’s wishes integrated	4.4 (0.6)
5	Reflective	Compatible with professional identity	4.3 (0.7)
6	Reflective	Goals personally meaningful	4.3 (0.6)
7	Reflective	Complements therapeutic process	4.1 (0.7)
8	Reflective	Children possess resources and strengths	4.1 (0.7)
9	Reflective	Interested in SFC goal-setting	4.1 (0.7)
10	Automatic	Feel positive using	3.9 (0.7)
11	Automatic	Motivated to use	3.8 (0.9)
12	Reflective	Makes sense to clinical team	3.8 (0.8)
13	Automatic	Feel anxious using	3.8 (0.9)
14	Reflective	Client’s goals often unrealistic	3.7 (0.7)
15	Automatic	Feel effective using	3.7 (0.7)
16	Automatic	Feel confident using	3.7 (0.7)
17	Automatic	Feel a loss of control using	3.7 (0.9)
18	Automatic	Feel intimidated using	3.6 (1.0)
19	Automatic	Organization supports	3.6 (0.9)
20	Automatic	I support use on team	3.6 (0.9)
21	Automatic	My team supports me	3.5 (1.0)
22	Automatic	Practice observed on team	3.3 (1.0)
23	Reflective	I direct client’s goals	3.3 (0.8)
24	Automatic	Unsure about practice	3.2 (0.9)
25	Automatic	Organization positively recognizes	3.1 (0.9)
26	Automatic	Client’s positively recognize	3.0 (0.9)
27	Reflective	Children’s goals sometimes unrealistic	2.8 (0.7)

^a^Higher ranking indicates higher perceived level of motivation by HCP with SFC goal-setting.

^b^All items scored from 1 to 5, where 5 indicates a most positive level of the attribute.

## Discussion

The most prominent barriers to HCP delivery of SFC goal-setting in paediatric rehabilitation were shown from this study to be HCPs low perceived competence in actual delivery of this care. These specific barriers were shown in tandem with general positive beliefs about SFC goal-setting, such as alignment with professional identity and motivation to set shared goals with children and families. HCPs positive beliefs about the value of SFC goal-setting were stronger than their perceived competence with setting the goals. This finding is consistent with literature on guideline implementation such as antimicrobial stewardship and surgical infections (Davey et al., 2015; Treadwell et al., 2014). In the situation of positive belief and low perceived competence, multifaceted targeted interventions such as: audit and feedback; use of local opinion leaders to model behaviour; supportive clinical decision support systems; coaching and mentoring; and team processes with the desired practice built into clinical routines, have demonstrated effectiveness (Davey et al., 2015; Grimshaw et al., 2002; McDonagh et al., 2018). Consistent with these clinical examples, use of the COMB in similar research indicates that support of ongoing skill and competency development are important to foster behaviour change (Michie et al., 2011, 2014).

In this survey, SFC goal-setting was not perceived to be integrated into clinical routines, clinical pathways, or the team culture. HCPs’ did not perceive that goal-setting was reinforced by clients, teams, or by the broader organization. HCPs may interpret this to mean that SFC goal-setting is not a valued practice and therefore lack ‘automatic-motivation’ to apply it. These barriers can be addressed through new approaches such as developing care pathways and plans that rigorously embed SFC goal-setting into clinical routines and team processes (Grimshaw et al., 2002). The practice can be supported with prompts, modelling or enablement (coaching and mentorship), and through use of audit and feedback to measure clinician performance and monitor fidelity (Michie et al., 2014).

The findings of this study reinforced previous literature findings that intervening at an organizational level is important to support practice change (Crawford et al., 2022). Perceived organizational barriers included a lack of organizational recognition for those who conduct these goals, as well as adequate time and resources to deliver it. These barriers could be addressed through environmental restructuring including additional and ongoing training for clinicians and explicit education and preparation for clients along with changes to clinician productivity targets and a recognition program for this practice (Crawford et al., 2022; Michie et al., 2011, 2014). Development of a SFC goal-setting champion network would provide social and practical support and facilitate problem-solving and action planning within teams, encouraging self-monitoring behaviours (Michie et al., 2011, 2014).

### Study limitations

A convenience sample of participants might not have been representative of all eligible HCPs. A significant number of participants were pursuing advanced SFC certification and may have biased the results. This potential bias is somewhat mitigated by the broad representation of health care disciplines that participated in the survey which are an adequate representation of the paediatric rehabilitation workforce.

The survey had a notable number of participants that filled in the demographic section of the survey but did not proceed. This might have been due to the time pressures on clinicians associated with workload, such as the shift to virtual care, during the COVID-19 pandemic.

### Implications for practice

Training providers to set collaborative goals with families using SFC in the form of workshops is insufficient to ensure they are confident to fully implement the approach. Organizational supports such as coaching HCPs, embedding goal-setting documentation in routine care, audit and feedback, and recognition for providers who integrate collaborative goals into their practice, are likely needed for full SFC implementation or other forms of child and family goal-setting to take root in paediatric rehabilitation.

## Conclusion

Paediatric rehabilitation HCPs view the provision of SFC goal-setting with clients and families positively but perceived that their individual competence to set collaborative goals was more of a challenge. Additionally, they perceived that they required more organizational supports to implement the approach fully. The survey based on the COM-B framework revealed which provider difficulties should be initial targets of future interventions. These barriers included; (i) difficulties with individual skills or experience to deliver SFC goal-setting (Capability) and, (ii) lack of clinical and team processes, support, time, and resources (Opportunity). Future implementation of SFC should target clinicians’ skills and competence to set collaborative goals as well as the best methods for introducing team and organizational support for this form of practice.

## Supplemental Material

Supplemental Material - Using the capability, opportunity, and motivation model of behaviour to assess provider perception of implementing solution-focused goal-setting in paediatric rehabilitation Supplemental Material for Using the capability, opportunity, and motivation model of behaviour to assess provider perception of implementing solution-focused goal-setting in paediatric rehabilitation by L Crawford, H Colquhoun, S Kingsnorth, D Fehlings, and Nora Fayed in Journal of Child Health Care
