# Supplementary material for: Using the capability, opportunity, and motivation model of behaviour to assess provider perception of implementing solution-focused goal-setting in paediatric rehabilitation
Source: J Child Health Care. 2023 Aug 16;29(2):311–23. doi: 10.1177/13674935231194501 (PMC12145463; doi:10.1177/13674935231194501)
Supplement: Supplemental Material - Using the capability, opportunity, and motivation model of behaviour to assess provider perception of implementing solution-focused goal-setting in paediatric rehabilitation [file sj-pdf-1-chc-10.1177_13674935231194501.pdf]

Supplement A – Capacity, Opportunity and Motivation (COM-B) Survey Items

COM-B Component	Survey Item Content
Capacity	Clinician focuses goals on real world activities
Capacity	Clinician has knowledge of SFC and its use in practice
Capacity	Formulates goals based on child's concerns
Capacity	Formulates goals based on client's desired future
Capacity	Activity/play based goals are set
Capacity	Formulates goals based on parent's concerns
Capacity	Formulates goals based on parent's desired future
Capacity	Clinician knows how to conduct SFC goal-setting practice with parents.
Capacity	Clinician understands assessment components.
Capacity	Understands intervention components.
Capacity	Is proficient in SFC goal-setting with clients
Capacity	Is competent in SF goal-setting with parents
Capacity	Is competent with clients
Capacity	Is adequately trained in SFC goal-setting with parents
Capacity	Is adequately trained with clients
Capacity	Is confident using SFC goal-setting with children
Capacity	Client goals based on past problems
Opportunity	SFC goal-setting improves child's outcomes
Opportunity	Organization supports SFC goal-setting
Opportunity	SFC goal-setting improves outcomes for parents
Opportunity	Colleague's support SFC goal-setting
Opportunity	Appropriate space is available for this practice.
Opportunity	Families support SFC goal-setting
Opportunity	Organization expects clinicians to use SFC goal-setting
Opportunity	There are adequate human resources to support this practice.
Opportunity	Team expects use of SFC goal-setting.
Opportunity	Documentation supports SFC goal-setting
Opportunity	Team has adequate time for, and uses SFC goal-setting for communication/coordination
Opportunity	Adequate team processes exist to support SFC goal-setting
Opportunity	Client and family expect to experience SFC goal-setting in practice
Motivation	Clinician listens to the child's wishes.
Motivation	Clinician believes children are experts in lives
Motivation	Client wishes are integrated into rehabilitation intervention
Motivation	Parent wishes integrated into rehabilitation intervention
Motivation	SFC goal-setting is compatible with professional identity
Motivation	Goals are personally meaningful to clients
Motivation	SFC goal-setting is complementary to therapeutic process

Motivation	Clinicians believe that clients have resources and strengths
Motivation	Clinician is interested in SFC goal-setting
Motivation	Feels positive using SFC goal-setting
Motivation	Is motivated to use it
Motivation	SFC goal-setting makes sense to team
Motivation	*Clinician feel anxious when using
Motivation	*Clinician believes that client goals often unrealistic
Motivation	Clinician feels effective using
Motivation	Clinician is confident using SFC goal-setting
Motivation	Clinicians feel a loss of control using
Motivation	Feels intimidated using
Motivation	Organization supports me to use SFC goal-setting
Motivation	I support team to use
Motivation	Team supports me to use SFC goal-setting
Motivation	Team observed using SFC goal-setting
Motivation	I direct my client's goals
Motivation	*Unsure about SFC goal-setting and what it is
Motivation	Organization positively recognizes me for this practice
Motivation	Clients positively recognize me using this practice
Motivation	*Client goals are sometimes unrealistic

*Reverse scored items so that a high score is consistently a positive COM-B component

Capacity includes Psychological and Physical subcomponents

Psychological Capability includes:

1) Knowledge, which means the HCP is aware of how to do SFC goal setting and they know the content and objectives of SFC goal setting
2) Memory, attention/decision processes, which means HCPs remember to use SFC goal setting and they are able to concentrate and focus attention on it.

Physical Capability includes:

1) Skills, which mean HCPs are trained and proficient in SFC goal setting.

Opportunity includes Social and Physical subcomponents

Social Opportunity includes:

1) Social Influences, which are those important to HCPs (team, leaders, profession) that support SFC goal setting practice.

Physical Opportunity includes:

1) Environmental Context and Resources, such as sufficient support (policies, processes, time, staff, space) for SFC goal setting

Motivation includes Reflective and Automatic subcomponents

Reflective Motivation includes:

1) Professional Role Identity, meaning HCPs feel that SFC goal setting is part of their work responsibility and consistent with their profession.
2) Beliefs about capabilities, meaning HCPs are confident that they could deliver SFC goal setting if they wanted, even if the client/caregiver is not motivated.
3) Optimism, meaning HCPs are optimistic about client goals and the future
4) Beliefs about consequences, meaning HCPs believe it benefits/has disadvantages for clients/caregivers
5) Intentions, meaning HCPs make a conscious decision to provide SFC goal setting

Automatic Motivation includes

1) Reinforcement, which are rewards, incentives, punishments whether they are/are not provided
2) Emotions, which represent the emotional state of an HCP
